# Pathology Applied

**Published:** 1884-07

**Authors:** Julius Schmidt

**Affiliations:** Rochester, N. Y.


### CLINICAL BUREAU.

### PATHOLOGY APPLIED.

Julius Schmitt, M. D., Rochester, N. Y.

[*Read before the Central New York Homœopathic Society.*]

Pathology means the study of the lesions or tissue-changes which disease causes in a healthy human body, and stands, therefore, in opposition to anatomy or the study of the normal condition of the tissues and organs of the human body. Lately, however, the term pathology has been extended, and especially by our school of medicine, to the study of the consequences following these lesions and the perverted action of diseased tissues and organs. The symptoms, subjective and objective, arising from these abnormal conditions have been grouped, and a name more or less appropriate has been given each group. Medical science, as taught by the old school, believes that the distinction between these different groups is the prime necessity for a successful treatment of the sick. After a given case has been diagnosed, treatment with them becomes entirely empiric, changing according to the individual fancies of so-called "authorities" or to the whimsical intuitions, if as such they might be honored, of the practitioner.

Basing therapeutics in the value of a diagnosis in the above sense has, as we all know, turned a good many homœopathic physicians from the road of success.

These erring brethren, imagining that these proceedings make them highly "scientific" and peers to the allopathic physicians to whom they look up with an awe-stricken heart, forget entirely that the aim of the true healer is to cure disease and not to write scientific certificates of death. An illustration of the success of this pseudo-science can be found in the "Fatal Case of

Metro-Peritonitis Following Labor," by J. W. Dowling, M. D., page 433 of the *Transactions of the American Institute of Homœopathy of* 1882.

This perverted use of pathology, however, must not interfere with its study, for we have to diagnose our cases as best we can, with all the means which this auxiliary branch of medical science offers, for the following reasons:

*First.* To distinguish between the symptoms properly belonging to the disease and those peculiar to the patient *whom we have to treat.*

*Second.* To form a prognosis.

*Third.* To direct the proper hygienic and dietetic measures.

*Fourth.* To recognize whether our patient is growing better or worse.

For all our pathological knowledge we have been indebted so far to our allopathic brethren, but lately men in our ranks have come forward, and among them Dr. R. R. Gregg, of Buffalo, has made the most startling announcement in regard to the character of diphtheria and other diseases, claiming bacteria, bacilli, micrococci, etc., to be nothing else but different stages of coagulated fibrin.

The question is, of course, an open one; but let us see whether Gregg's views help us as practical physicians. The best answer can be given by the following illustration from the green tree of Practice "for [according to Goethe] all theory is gray!"

On the 26th of December, 1881, I was called to see Mary H., three years old, a blonde, sparely built girl, who had been exposed to diphtheria by her mother's visiting a neighboring house where a child died with diphtheritic croup. She complains of being tired, of backache. There is a purplish appearance of the mucous membranes of the lips, lower one a little swollen, of buccal cavity and throat; a diphtheritic spot not larger than a pin's head on the left tonsil; tongue was pointed and purplish; pulse weak, 132; skin hot; urine scanty, dirty-colored, and containing albumen; bowels constipated; in fact, the case was of that adynamic sort which we all dread to meet. Lachesis$^{cm}$ (Swan), one dose, dry on tongue, Placebo in water.

December 27th, 1881.—Child seems somewhat livelier; pulse 120; no other change; continue Placebo.

December 28th.—No change; pulse 128. Lachesis$^{cm}$, one dose dry, and Placebos.

December 29th.—No change; rather weaker; pulse 132.

Now Lachesis was evidently indicated and must be given, but how? Give the cm potency dissolved in water every two or three hours. But I had seen such dreadful results from repetitions even in this way that I did not dare to do that. The higher potency, however, has always in my experience acted stronger than the lower one, and therefore I gave Lachesis$^{mm}$ (Swan), one dose dry, and Placebos in water.

December 30th.—When I entered the house this morning the mother received me with the greatest alarm, and the condition of the child was such as to alarm anybody. Submaxillary glands on both sides fearfully swollen; running of acrid secretion from nose and mouth; the famous diphtheritic stench; the throat and even lips covered with thick, gray membranes. I must confess I was stupefied at first, but rallying, I asked the mother, Well, how is the urine? "Oh! she made plenty of that, and her bowels moved also." A centweight fell from my heart. More favorable symptoms were found in the color of the water, which was clearer, and also in the pulse, which had fallen to 120 beats in the minute. I quieted the mother; told her the child would be better to-morrow, for I reasoned, according to Gregg, that the superabundant fibrin in the blood had found its normal outlet, and that the metastasis of the disease from the kidneys to the throat was certainly a step for the better. Sacch. lactis. in water.

December 31st.—Child is in her little chair, busily employed with her dolls and playthings; part of the diphtheritic membranes were hanging loosely in the throat, ready to drop off; stench gone; acrid discharge much less; patient wants something to eat; pulse 96. In a few days she was entirely well and has not been sick since.

So much for the pathology of diphtheria as taught, *not* by the "*awe-inspiring, regular* SCIENTIFIC physician," but by R. R. Gregg, M. D., nothing but an humble follower of the great master, Hahnemann.

How the micrococci, vibriones, bacilli, and the other horrible little monsters got killed so quickly I do not know, but they certainly could not stand the Lachesis in the mm potency.

